# Metastatic Breast Cancer: Review of Emerging Nanotherapeutics

**DOI:** 10.3390/cancers15112906

**Published:** 2023-05-25

**Authors:** Ranga Dissanayake, Rheal Towner, Marya Ahmed

**Affiliations:** 1 Department of Chemistry, University of Prince Edward Island, 550 University Ave., Charlottetown, PE C1A 4P3, Canada; radissanayake@upei.ca (R.D.); rheal.towner@upei.ca (R.T.); 2 Faculty of Sustainable Design Engineering, University of Prince Edward Island, 550 University Ave., Charlottetown, PE C1A 4P3, Canada

**Keywords:** breast cancer, metastasis, nanotherapeutics

## Abstract

**Simple Summary:**

Breast cancer that spreads to other parts of the body, known as metastatic breast cancer, is a serious and life-threatening condition, leading to decreased survival. Current treatment options for metastatic breast cancer are similar to those used for the initial cancer, including surgery, chemotherapy, immunotherapy, and radiotherapy. However, metastatic breast cancer is more complicated because cancer cells in different organs behave differently, making it harder to treat effectively. To address this challenge, nanotechnology in combination with current cancer therapies can be used. Nanotherapeutics involves using tiny particles to deliver therapies directly to cancer cells. This approach has shown promise in both primary and metastatic breast cancer treatments, with new ideas and technologies constantly being developed. This review article provides detailed information about the recent progress and future possibilities of using nanotherapeutics specifically for the treatment of metastatic breast cancer. It takes into account the unique characteristics of the disease, as well as discusses how nanotechnology can be combined with existing treatments. It also explores the potential for these combined approaches to be used in future clinical settings.

**Abstract:**

Metastases of breast cancer (BC) are often referred to as stage IV breast cancer due to their severity and high rate of mortality. The median survival time of patients with metastatic BC is reduced to 3 years. Currently, the treatment regimens for metastatic BC are similar to the primary cancer therapeutics and are limited to conventional chemotherapy, immunotherapy, radiotherapy, and surgery. However, metastatic BC shows organ-specific complex tumor cell heterogeneity, plasticity, and a distinct tumor microenvironment, leading to therapeutic failure. This issue can be successfully addressed by combining current cancer therapies with nanotechnology. The applications of nanotherapeutics for both primary and metastatic BC treatments are developing rapidly, and new ideas and technologies are being discovered. Several recent reviews covered the advancement of nanotherapeutics for primary BC, while also discussing certain aspects of treatments for metastatic BC. This review provides comprehensive details on the recent advancement and future prospects of nanotherapeutics designed for metastatic BC treatment, in the context of the pathological state of the disease. Furthermore, possible combinations of current treatment with nanotechnology are discussed, and their potential for future transitions in clinical settings is explored.

## 1. Background

Breast cancer (BC) is the most commonly occurring type of cancer in women worldwide. Among different cancer forms, BC is one of the most heterogeneous types, differing significantly among different patients (intertumoral heterogeneity) and within each individual tumor (intratumor heterogeneity). BC becomes more lethal and complicated during advanced and metastatic stages [1]. According to the statistics, 20–30% of BC patients may obtain metastases soon after diagnosis and primary tumor treatment [2,3]; in certain cases, metastasis of BC cancer can be found even before the diagnosis of primary tumor is made [4]. Metastasis of BC is mostly observed in bones, liver, lungs, and brain, and its spread to other visceral organs is rare. Metastasis of BC presents a significant challenge in assessing and battling cancer, because metastatic cells are able to invade to different organs, and complex tumor cell heterogeneity, plasticity, and a distinct tumor microenvironment (TME) collectively modulate the response against treatment [5]. The most common BC metastatic site is bone, which accounts for nearly 75% of metastatic cases [6], with an overall 5 year survival rate of 22.8% [7]. The second most frequent site of BC metastasis is lung, while liver is the third. Nonetheless, the 5 year survival rate of liver metastasis is lower than both bone and lung [2]. Around 20% of patients with metastatic BC tend to develop brain metastases, which results in both poor life expectancy and living quality in many patients. The median overall survival (OS) following diagnosis of brain metastases from BC is indicated as 7.9 months. Overall, metastasis of BC is reported to be associated with approximately 90% of cancer-related deaths, and current treatment regimens contain several deficiencies that limit their desirable outcomes [7,8]. Therefore, there is an urgent and pressing need to develop and discover new therapeutic approaches to combat the disadvantages associated with conventional therapies.

Nanotechnology has shown promising results in various biological applications including diagnosis and breast cancer treatment. As a theragnostic platform, nanotechnology has the capability to precisely target cancerous tissue and deliver the drug payload with minimal side-effects. The inherent nature of nanomaterials can enhance cell penetration efficacies and self-targeted accumulation of nanoparticles to the desired site. However, much work is required to utilize nanotherapeutics as reliable platforms for cancer treatment. The applications of nanotherapeutics for both primary and metastatic BC treatments are being discovered rapidly, and new ideas and technologies are being developed. The development of nanotherapeutics, as a function of BC subtypes and heterogeneity for primary BC treatment, is discussed elsewhere in detail [9,10,11]. The focus of this review is to provide comprehensive details on the recent advancement and future prospects of nanotherapeutics designed for metastatic BC treatment. 

## 2. Metastatic Breast Cancer

### 2.1. Nature and Pathophysiology

According to the cancer statistics, around half of patients display clinically detectable metastatic disease when diagnosed [3]. Furthermore, patients without metastasis at the time of diagnosis possess a high probability of having micro-metastasis sites that cannot be detected using conventional detection techniques [2]. Hence, metastasis is the most life-threatening consequence for patients diagnosed with cancer. The metastatic process comprises three distinct stages: invasion, intravasation, and extravasation. Through the initial invasion stage, the BC cells achieve an aggressive attribute that permits them to migrate and occupy the basement membrane, subsequently leaving the primary tumor site. Cancer cells undergo several invasion strategies, including mesenchymal migration, ameboid migration, chain migration, and collective/cluster migration [12,13,14].

Mesenchymal and chain migrations are the most abundant strategies for the invasion of breast metastasis [12]. In mesenchymal migration, tumor cells go through epithelial-to-mesenchymal transitions (EMT) to decrease their tight junctions and to enter adjacent capillaries or lymph vessels. EMT is depicted by the loss of epithelial traits and adaption of mesenchymal characteristics, which offers migratory potential [15]. Initiation of EMT involves the loss of cell–cell adhesions, alterations in the expression of specific cell-surface proteins, activation of transcription factors, generation of ECM-degrading enzymes, and reorganization and expression of cytoskeletal proteins. EMT is portrayed by the combined loss of epithelial cell junction proteins, consisting of E-cadherin, α-catenin, claudins, occluding, and ZO-1. During EMT, there is an amplified expression of mesenchymal markers, including as *N*-cadherin, vimentin, and fibronectin, along with reorganization of the cytoskeleton, which collectively results in the loss of apical–basal cell polarity and the attainment of a spindle-shaped morphology [15,16,17,18].

Entrance of tumor cells into the circulation (intravasation) and their exit from the circulation (extravasation) to a host tissue represent critical phases in the metastatic process. A clear contrast between intravasation and extravasation is the makeup of the blood vessels. At the intravasation site, tumor blood vessels are irregular and malformed, frequently possessing breaks in their thin lining that allow easy access of tumor cells into the circulation. However, the vasculature of normal tissue, where tumor cells extravasate, do not have these same characteristics [19]. Micro-vessel systems with strong permeability sinuses in some organs, such as bone marrow and liver, facilitate the cancer cell migration to the target organ. However, in many other organs, including lungs and brain, the endothelial cells form a continuous barrier that blocks the direct penetration of cancer cells [20,21]. In such instances, white blood cells and platelets help tumor cells traverse the vasculature by creating complexes through L- and P-selectin. As such, overexpression of selectin ligands on the surface of tumor cells is correlated with metastatic progression and poor prognosis. Chemokines secreted by target tissue can also induce directed cell migration, by initiating cell signaling pathways and by monitoring cytoskeletal rearrangement and adhesion [22]. Apart from blood circulation, the lymphatic system also plays a significant role in metastasis. Tumor cells initially breach the basement membrane of the epithelium and actively enter the lymphatic vessels. Subsequently, they establish themselves within lymph nodes, leading to lymphatic metastasis, which serves as an early indicator of distant organ metastasis [23].

BC metastasis has a direct connection with the primary tumor, and different BC subtypes exhibit unique metastatic site preferences, a phenomenon termed organotropism of metastasis [19]. All BC subtypes have the ability to develop bone metastasis; however, luminal subtypes A and B exhibit a higher bone metastasis rate (80.5%) as the first metastatic site, when compared with HER-2 positive (55.6%) and basal-like (41.7%) BCs [24]. Luminal B and basal-like subtypes display a higher risk of lung-specific metastasis, while the HER2-negative subtype and HER2-positive subtypes frequently result in liver metastases. Brain metastasis is common with basal-like and HER-2-positive tumors [25]. 

The bone, lung, liver, and brain are frequently targeted organs for breast cancer metastasis, in addition to distant lymph nodes. Among these four primary sites of metastasis, bone and lung metastasis are prevalent occurrences, accounting for approximately 60% of all breast cancer metastases [19,24]. Liver metastasis represents around 7% of cases. Despite this, conventional therapies such as chemotherapy, radiotherapy, and surgery remain the most effective options, with only limited research conducted on nanotherapeutic approaches for liver metastasis [26]. Although brain metastasis is less common in breast cancer patients, the disease causes progressive neurological disability, and the lack of effective treatments for brain metastatic BC at present is concerning [27]. Consequently, there is a significant need to discover alternative and effective treatments for the metastatic BCs. Therefore, this review primarily focuses on nanotherapeutic approaches for bone, brain, and lung metastasis of breast cancer. 

### 2.2. Current Treatment Regime for Metastatic BC and Drawbacks

Systemic drug therapies such as chemotherapy, targeted drugs, hormone therapy, immunotherapy, and combinatorial therapies are the foremost treatment options for women diagnosed with stage IV breast cancer [28]. Surgery and radiotherapy are also useful depending on the tumor stage and location. Hormone receptor positive (ER^+^/PR^+^) metastatic BC is typically treated with hormone therapy (tamoxifen/aromatase inhibitor), and an amalgamation of hormone therapy with targeted drugs such as a cyclin-dependent kinase 4/6 (CDK4/6) inhibitor, everolimus, and phosphoinositide 3-kinase (PI3K) inhibitor is shown to improve the effectiveness of monotherapy. The combination of chemotherapeutics with anthracyclines or taxanes is an example of an effective treatment for metastatic BC [29]. HER2-positive cancers are treated with chemotherapy combined with trastuzumab (Herceptin) and pertuzumab. As alternatives, antibody–drug conjugates are used to obtain targeted delivery of drugs with a dual mechanism of action. Trastuzumab deruxtecan (Enhertu) is a recently approved antibody–drug conjugate that is being used as a second line of treatment for HER2-low metastatic BC. BRCA-mutated TNBCs are typically treated with poly ADP ribose polymerase (PARP) inhibitors, such as olaparib or talazoparib. Hormone therapies and chemotherapeutics are also very beneficial in treating BRCA-mutated cancers [30,31].

PI3K CA mutation is common among the metastatic ER-positive breast cancers and can be treated with PI3K inhibitors such as alpelisib [32]. PI3K inhibitors, combined with the hormone drug fulvestrant, are used to treat postmenopausal women with advanced ER^+^/PR^+^ BCs [33]. A combination of immunotherapy and chemotherapy is used for advanced triple-negative breast cancer (TNBC) treatments, bearing programmed death ligand-1 (PD-L1) protein [34,35].

Similar to primary BCs, repetitive treatments of metastatic BCs with chemotherapeutics lead to treatment failure and the emergence of chemotherapeutic resistance [36]. At this time, there is no universal monotherapy or combination palliative chemotherapy for patients who are pretreated with anthracyclines or taxanes [37]. The median survival rate of patients with BC metastasis is within the range of 3 years. Accumulating toxicity of chemotherapeutics is the most significant challenge with metastatic BC treatment. Ongoing emerging resistance leads to the use of different therapeutics in sequence, which results in exposing patients to various toxicities. A lack of facilities and the high costs associated with novel biomarker studies are the other main problems associated with the conventional treatment regimen for metastatic BC [29,38]. The development of multifunctional nanotherapeutics has the substantial potential to fill the existing gap in the metastatic BC therapeutic field. In the field of cancer management, nanomedicines have demonstrated enhanced permeability and retention of drugs, which has resulted in effectively targeting diseased tissues and reducing systemic toxicities [39]. Lipid-based nanodrugs (Doxil^TM^, Caelyx^TM^, Myocet^TM^, DaunoXome^TM^, Mepact^TM^, Ameluz^TM^, Marqibo^TM^, Onivyde^TM^, and Vyxeos^TM^), protein–drug conjugates (Oncaspar^TM^, Eligard^TM^, Abraxane^TM^, Pazenir^TM^, and atezolizumab/nab-paclitaxel) and metallic nanoparticles (NanoTherm^TM^) are FDA/EMA-approved drug formulations which are already in the clinical setting for cancer treatments [39,40,41,42]. Therefore, the advancement of nanotherapeutics can provide improved efficiency of the drug with fewer toxicity concerns for metastatic BC treatment.

In nanotechnology, developing a favorable nano-formulation entails numerous physical, chemical, biological, and functional properties for cancer treatment. Unlike primary cancer treatment, metastatic treatments are complex, requiring simultaneous delivery of drugs at both primary and metastatic sites. In general, metastatic sites of BC tend to be physiologically diverse than the primary site. In addition, molecular subtyping of the primary tumor is often not repeated at the metastatic site, and the metastasized tumor possesses markers identical to the primary tumors. Therefore, nanomaterials that are used to target metastasis should have distinct functional and structural features with ubiquitous properties. Properties that include high drug loading efficiency, retention in the tumor microenvironment, an extensive half-life in circulation with minimal systemic toxicity, selective localization, enhanced internalization into the tumor via endocytosis, sustained and controlled release properties, and effective bioelimination from the body are key attributes of nano-formulations for metastatic BC treatment [43,44,45]. 

## 3. Nanotherapeutics for Breast Cancer Bone Metastasis

**Figure 1:**
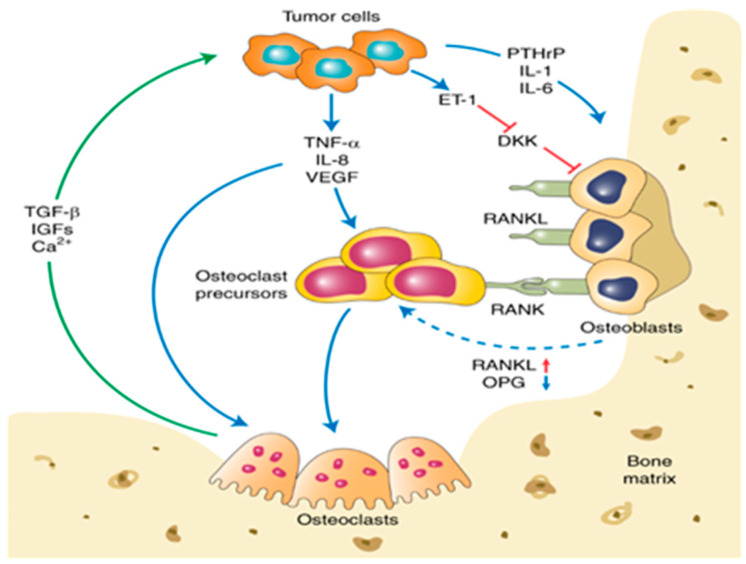
The vicious cycle between BC cells and bone. Tumor cells interact with both osteoclasts and osteoblasts in the bone microenvironment, leading to a local increase in tumor-derived factors that promote osteoclastogenesis and osteoblastogenesis. Mature osteoclasts subsequently release survival factors that include insulin-like growth factor 1 (IGF-1) and transforming growth factor beta (TGF-β), which promote the proliferation and survival of tumor cells [51].

The incremental process of bone metastasis development is initiated by establishing a premetastatic niche at distant organs created by several tumor-derived factors (CCL2, IL6, lysyl oxidase (LOX), and Dickkopf WNT signaling pathway inhibitor 1 (DKK1)) of the primary tumor. Disseminated tumor cells enter the bone via parenchymal tissue by degrading the vascular basement membrane of blood and lymph vessels. Bone metastases are categorized as either osteolytic or osteoblastic (osteosclerotic), while osteolytic metastasis lesions are the dominant type. Upon entry into the bone microenvironment, tumor cells change their biological phenotype to an aggressive osteolytic phenotype [46,47]. Furthermore, tumor cells can survive and initiate their growth in a bone microenvironment due to the upregulation of several signaling pathways of tumor cells, including WNT, NF-κB, and CREB. Several cytokines and chemokines that are typically produced by bone cells to support bone homeostasis also foster the growth of tumor cells within bone tissues. For example, osteoblasts generate osteoprotegerin (OPG), a decoy receptor-to-receptor activator of NF-κB ligand (RANKL) that curtails osteoclast activation. RANKL plays a vital role in bone resorption but can also prompt bone metastasis by recruiting RANK-expressing cells directly to the bone [47,48,49]. RANK, RANKL, C–X–C motif chemokine receptor type 4 (CXCR4), and CXCL12 are of great importance for homing BCCs to the bone. Meanwhile, the release of essential tumor growth factors, such as transforming growth factor β (TGF-β), VEGF, IGFs, and BMPs, by tumor cells activates osteoclasts and stromal cells, as well as promotes angiogenesis and tumorigenesis [47,50]. Extravasated tumor cells to bone may grow immediately or enter a dormant state that may last for years, and they can be reactivated at any timepoint to create metastasis. The detailed molecular mechanism of BC bone metastasis is provided in Figure 1 [51].

Bone metastasis is a very common phenomenon for all types of BC including ER^+^ and TNBCs, and 85% of the cases are associated with osteolytic lesions that limit the life span of the patients by several years [52]. The osteolytic lesions also reduce the quality of life by causing bone fractures, spinal cord compression, and hypercalcemia. Bone metastasis of BC can be treated in two possible ways: (i) by inhibiting the growth of cancer cells, and (ii) by reducing the activity of osteoclasts. However, chemotherapy is thus far the main and primary option as the baseline treatment for bone metastasis [52,53]. The poor blood supply to bone tissue drastically reduces the efficacy of chemotherapies, thus producing enhanced dose dependent toxicities [54]. Therefore, it is necessary to introduce bone-targeted drug delivery systems (DDSs) for inhibiting the growth of cancer cells and for reducing the osteoclast activity of bone [48]. Targeted DDSs play a vital role in payload delivery to the metastatic site, thereby increasing the treatment efficacy and reducing the adverse effects.

Hydroxy apatite (HA) and ανβ3 integrin are likely targets for BC bone metastasis. Tetracycline, bisphosphonate (BP), certain amino acids (aspartic acid), carboxylated glutamic acids (Gla), and aptamers are being investigated as targeting agents due to their high affinity for bone tissues [55,56]. However, arginine–glycine–aspartic acid (RGD) peptide and alendronate (ALN) and zoledronic acid from the bisphosphonate family are the most frequently used bone-targeting agents [56,57]. 

### 3.1. Alendronate Functionalized Nanocarriers

Alendronate, a second-generation BP has been used to target BC bone metastasis. Several nanotherapeutic systems decorated with ALN have been developed, incorporating various chemotherapeutic agents. For instance, He et al. developed an alendronate-decorated phospholipid coating with ultrasmall coordination polymer nanoparticles (CPNs) of ~55 nm in diameter. CPNs were loaded with the prodrug of cisplatin, cis,cis,trans-diammine-dichloro-disuccinato-platinum (DSP) and Zn^2+^ metal ions. The nanoparticles were prepared by mixing polymers, prodrug, and Zn^2+^ ions in a water-in-oil (W/O) microemulsion, and then stabilized with a phospholipid coating [58]. The nanoparticles formed were decorated with alendronate PEG conjugates, prepared using a fatty acid (ASA-c18) linker between PEG and ALN PEG. Hence, the system contained (I) DSP and Zn-loaded biodegradable CPNs in the form of a rigid inner core, (II) an outer stealth layer of PEG to extend the blood circulation time, and (III) alendronate as a targeting moiety. The outcomes of this study demonstrated that the developed system showed ~4-fold higher drug accumulation in bone metastatic lesions compared to healthy bone tissue. Furthermore, tail vein administration of DSP-Zn@PEG-ALN NPs to MDA-MB-231 tumor-bearing mice effectively inhibited tumor growth and showed decreased osteocalastic bone destruction. DSP-Zn@PEG-ALN NPs showed significantly diminished toxicity of cisplatin; interestingly, researchers assessed the analgesic property of the system that helps to mitigate the cisplatin-induced toxicity-related pains. In vivo pharmacokinetic studies indicated that DSP-Zn@PEG-ALN NPs could significantly extend the blood circulation time, an ideal attribute for the storing of nanoparticles into bone tissue [58].

ALN- and folic acid (FA)-conjugated PLGA nanoparticles termed D-α-tocopheryl polyethylene glycol succinate-conjugated (ALN-TPGS) and folic acid-conjugated (FA-TPGS) TPGS were investigated for paclitaxel delivery in TNBCs by Chen and coworkers [59]. ALN/FA-decorated dual-targeted nanoparticles are capable of selective affinity for hydroxy apatite in bone via ALN, and for overexpressed FA receptors in cancer cells via FA. Therefore, the developed targeted system significantly reduced PTX-related cytotoxicity. The results showed that the nano-system notably amassed in the bone metastatic site in vivo and reduced 4T1 primary tumor growth and its lung metastases, resulting in a significant improvement in the survival rate of the treated mice. The micro-CT images revealed that the 4T1 tumor-bearing mice treated with the ALN/FA-decorated PTX-loaded nanoparticles inhibited bone destruction and bone loss when compared with the controls, and minimized unwanted adverse effects of therapeutics on the normal tissues of the mice were observed [59].

Doxorubicin-loaded human serum albumin-based nanoclusters produced by ball-milling technology and decorated with ALN (HSA-AD/DOX) showed ~5-fold higher affinity compared to the HSA/DOX in the hydroxyapatite-containing heterotopic human osteosarcoma cell line (HOS/MNNG) in vitro, as well as in a mouse xenograft model. It was shown that the ball-milling method can preserve more than 90% of the secondary structures of albumin post nanoparticle fabrication. Furthermore, an in vitro bone cancer model was developed by coculturing hydroxyapatite and collagen, the key components of the bone matrix that represent a highly mineralized bone tumor microenvironment, with HOS/MNNG. HSA-AD/DOX also showed increased (52.0%) tumor accumulation compared to that of the unmodified HSA/DOX (45.1%) as a result of the hydroxyapatite-binding affinity of the ALN moiety. HSA-AD/DOX also changed the apoptotic and proapoptotic protein (TP53, caspase-1, caspase-3, caspase-9, and PARP) expression pattern of the tumor, thus enhancing the apoptotic process [60].

Curcumin is a well-known natural compound with potential anticancer activities. Therefore, curcumin-loaded nanomaterials for metastatic cancers exhibit added advantages over cytotoxic drug-loaded systems. Redox-sensitive alendronate targeted micelles loaded with curcumin (ALN-oHA-SS-CUR) developed by Dong et al. successfully inhibited the growth of MDA-MB-231 tumors both in vitro and in vivo. Stimulus-responsive nanoparticles were formulated via disulfide linkage between hydrophobic curcumin and hydrophilic oligosaccharide hyaluronan (oHA) and exhibited selective binding to the CD44 receptors of TNBCs. The drug release rate from the nanoparticles was found to be proportional to the concentration of glutathione, due to the oxidation of disulfide linkages, thereby disrupting the nanoparticle morphology [61].

**Figure 2:**
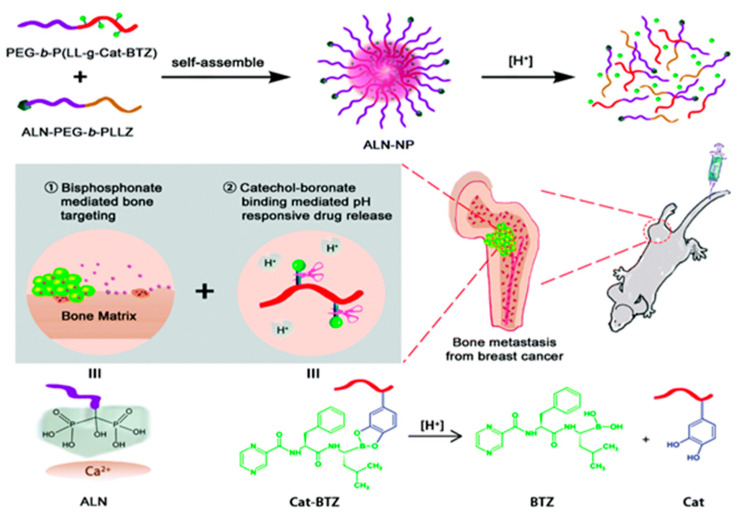
Schematic depicting the generation of ALN-NPs, as well as the process of combined bone-targeting and pH-responsive release of bortezomib resulting from the aryl boronate linkage as an antimetastatic therapy. ALN: alendronate; Cat: catechol; BTZ: bortezomib [54].

Another group developed alendronate-targeted nanoparticles composed of PLGA loaded with curcumin and bortezomib. Bortezomib is an anticancer agent used to treat multiple myeloma and mantle cell lymphoma. The dual drug delivery system showed enhanced accumulation and tumor shrinking ability with MDA-MB-231 tumor-bearing mice. The targeted nanoparticles could inhibit the bone resorption process. However, the combination of curcumin and bortezomib did not show any synergistic effects on the anti-osteoclastogenic activity of the bone [62]. Zhu et al. developed ALN-decorated, bortezomib–catechol prodrug-loaded multicomponent mixed micelles, via self-assembly of PEGylated block copolymers. The multicomponent micelles were synthesized by mixing PEGylated bortezomib–catechol prodrug with PEGylated ALN conjugates. The incorporation of an acid-labile aryl boric acid ester linkage between PEG and prodrug yielded pH-sensitive drug release in the tumor microenvironment. The detailed synthetic procedure of micelle development is depicted in Figure 2. In vitro and in vivo investigations demonstrated that, compared with free drugs or control micelles, the prodrug-loaded stimulus-responsive micelles (ALN-NP) exhibited decreased systemic toxicity and beneficial therapeutic impacts [54].

Bone-targeted lipid/oil based triptolide nanoparticles (TPNs) (90 nm) decorated with ALN developed by Wen and team showed promising anticancer activities against MDA-MB-231 breast cancer cells both in vitro and in vivo. The formulated lipid-based system showed 97% drug encapsulation efficacy and a biphasic controlled release profile for 5 days. TPNs were more cytotoxic against cancer cells but had decreased cytotoxicity against MC3T3-E1 osteoblasts. If combined with docetaxel or paclitaxel, low-dose TPN substantially increased the efficiency of the two chemotherapy drugs against MDA-MB-231, indicating potent chemosensitization effects [63].

ALN-conjugated polyethylene glycol, polyglutamic acid, and polyphenylalanine (PEG–PGlu–PPhA) copolymer micelles produced via nanoprecipitation were utilized for selective delivery of docetaxel against 4T1/Luc xenografts in mice. ALN-modified micelles produced sustained release, enhanced cytotoxicity, and improved pharmacokinetic abilities. Therefore, treatment with targeted DTX-loaded micelles resulted in the attenuation of tumorigenesis and significantly enhanced animal lifespan, as opposed to the conventional surfactant-based formulation (free DTX) [64]. A pH- and redox-sensitive doxorubicin-loaded ALN-conjugated, crosslinked PEG–hyaluronic acid-functionalized poly(aspartic acid)-based nanosystem (DOX@ALN-(HA-PASP)CL) was developed by Zhao and coworkers. The pH-sensitive linker shed ALN at reduced pH, in response to the protons released by osteoclasts and tumor cells, and the released ALN further inhibited the osteoclast activity of the tumor. The shedding of ALN exposed hyaluronic acid that facilitated the NP uptake by MDA-MB-231 tumor cells via CD44 receptor activation. DOX@ALN-(HA-PASP)CL showed relatively higher negative zeta potentials due to the presence of carboxyl groups on the HA backbone, thus preventing nanoparticle aggregation, precipitation, and adsorption of opsonin in blood [65].

Graphene oxide (GO) is a unique class of nanomaterial that has been recently explored as drug delivery carrier. Pham et al. developed ALN-functionalized doxorubicin-loaded GO nanosheets as nanotherapeutics for bone metastatic breast cancer treatment. In vivo biodistribution studies showed that ALN-targeted GO nanoparticles were retained longer and at increased concentrations in bone tumors compared to nonfunctionalized GOs. Their results suggest that ALN-functionalized GO could be used as a favorable carrier to improve antitumor effects and reduce the off-target effects of DOX for the treatment of bone metastasis [66].

Targeted chemo-photodynamic therapy was developed by alendronate-functionalized bortezomib zinc phthalocyanine NPs for BC bone metastasis. In vivo studies in MDA-MB-231 tumor-bearing mice showed that BTZ@ZnPc-ALN treatment under irradiation could reduce the tumor volume by 85% compared to the control group, via ROS-induced mitochondrial damage. In addition, excessive ROS-related endoplasmic reticulum stress post nanoparticle treatment was observed due to the expression of GRP78 protein and the increase in cytosolic Ca^2+^ levels, leading to the inhibition of tumor cell proliferation [67].

The transcription factor Gli2 drives the expression of parathyroid hormone-related protein (PTHrP) that activates osteoclast-mediated bone destruction. Blocking the activity of Gli2 is a favorable approach for the treatment of BC bone metastasis. An amphiphilic diblock copolymer of poly[(propylene sulfide)-block-(alendronate acrylamide-co-*N,N*-dimethylacrylamide)] [PPS-b-P(Aln-*co*-DMA)] was used to deliver GANT58, a small-molecule Gli2-inhibitor, to bone-associated tumors. In an intracardiac tumor cell injection model of BC bone metastasis, treatment with the lead candidate of the GANT58-loaded polymeric NP formulation diminished the tumor-associated bone lesion area threefold and enhanced the bone volume fraction in the tibiae of the mice 2.5-fold. These results suggest that GANT58-loaded polymeric NPs have the capability to reduce tumor-driven osteoclast activation and subsequent bone destruction in patients with bone-associated tumor metastases [68].

### 3.2. Zoledronic Acid-Functionalized Nanocarriers

Zoledronic acid (ZLA), a third-generation BP, has been used for treating bone resorptive diseases, including osteoporosis and osteoporotic fractures. The administration of zoledronic acid substantially reduced the pain and improved the quality of life among BC bone metastasis patients. Due to a high affinity and strong targeting potency of ZLA toward bone, the drug has been used as a targeting agent in DDSs to treat BC bone metastasis [69]. The first research on ZLA targeting for BC bone metastasis was published in 2012 and demonstrated the use of PEGylated polybutyl cyanoacrylate (PBCA) NPs functionalized with ZLA to deliver docetaxel to bone metastasis site [70]. Thereafter, several researchers focused on evaluating the efficacy of ZLA as a targeting agent for bone metastasis, and the key findings are summarized below. 

**Figure 3:**
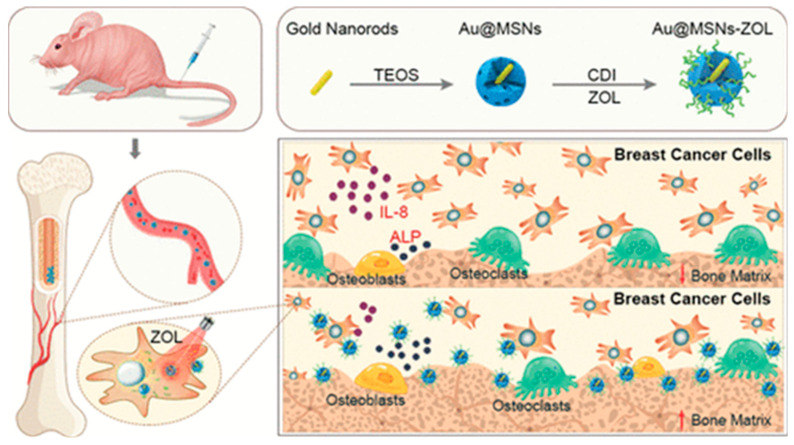
Bone-targeted nanoplatform combining zoledronate and photothermal therapy in order to treat breast cancer bone metastasis. The system contains gold nanorods enclosed inside zoledronic acid-functionalized mesoporous silica nanoparticles (Au@MSNs) [53]. (Reprinted/adapted with permission from Ref. [53]. 2023, Marya Ahmed).

Sun and team synthesized gold nanorod-loaded mesoporous silica nanoparticles (Au@MSNs) that were subsequently conjugated with ZLA as photothermal therapy agents for bone metastasis. The nanoparticles (Au@ MSNs-ZLA) demonstrated bone-targeting ability in vivo, which resulted in the inhibition of the formation of osteoclast-like cells, and subsequently enabled osteoblast differentiation in vitro. Combined Au@MSNs-ZLA and photothermal therapy (PTT), which was triggered by near-infrared irradiation, decreased tumor growth both in vitro and in vivo, and diminished pain and bone resorption in vivo by inducing apoptosis in cancer cells, as well as improved the bone microenvironment. This single nanoplatform combined ZLA and PTT to provide an exciting strategy for breast cancer bone metastasis treatment. The findings of this study are summarized in Figure 3 [53].

Superparamagnetic iron oxide (Fe_3_O_4_) and indocyanine green (ICG)-entrapped PLGA nanoparticles, modified by ZLA (PLGA-ZLA) NPs (ICG/Fe_3_O_4_@PLGA-ZLA), were developed for breast cancer tibial metastasis treatment. In this system, both ICG and Fe_3_O_4_ had photothermal properties, while ZLA-targeted Fe_3_O_4_-embedded NPs were attracted to the medullary cavity of the tibia under an external magnetic field. Dual-targeted and double photothermal agents containing NPs resulted in high accumulation in the tibia, as well as superior PTT efficiency. Additionally, in vivo studies demonstrated the extraordinary targeting and antitumor therapeutic effects of NPs, as well as their localization in the medullary cavity of the tibia, to resolve the difficulties with deep lesions associated with breast cancer tibial metastasis, demonstrating the great potential of these nanocarriers as cancer theranostics [71].

Pang and coworkers designed bone-targeting metal–organic framework (MOF) nanoparticles modified with ZLA to deliver immunostimulatory cytosine–phosphate–guanosine (CpG) motifs (BT-isMOF) in metastatic bone lesions. In vitro and in vivo studies showed the strong targeting capability of BT-isMOF nanoparticles to metastatic bone lesions, resulting in substantial reduction. The bone resorption was facilitated by osteoclasts, and induction of macrophage polarization to the proinflammatory (M1) phenotype post treatment was observed. The M1 phenotype of macrophages is recognized by the secretion of proinflammatory cytokines, and it plays a role in antitumor activities [72].

### 3.3. Other Nanocarriers

Tripeptide RGD-targeted pH-responsive dendrimers bearing a boronate–catechol linkage were developed by Wang and coworkers for the delivery of bortezomib to metastatic tumor sites. RGD targeting of DDSs showed successful delivery of the dendrimers to the TNBC cells. Moreover, the X-ray and micro-CT results confirmed that the bone destruction of the tibia in the RGD-targeted dendrimer-treated group was lower, and the bone volume and number of trabeculae were higher compared to the respective control groups. These studies proposed that RGD-targeted dendrimer nanocarriers could obliterate the osteolysis caused by BC cells [57].

Osteoclasts exhibit high expression of the β(3) integrin and can be used as an alternative targeting moiety for bone metastatic BC treatment. Phospholipid/polysorbate 80 micelles decorated with integrin-specific quinolone nonpeptide ligand (3-MPs) were developed to transport docetaxel prodrug to the metastatic bone sites. The 3-MPs-coated micelles showed about 6.5-fold higher affinity for the bone tissue compared to nontargeted micelles in vivo. Additionally, after the administration of 3-MPs/DTX-PD, the tumor volume and tumor-induced bone loss were substantially decreased, along with lower hepatotoxicity, when compared to the docetaxel-treated group [73].

## 4. Nanotherapeutics for Brain Metastasis

Compared to bone, lung, and visceral metastasis, brain metastasis is less common in breast cancer patients. However, the progressive neurological disability and the lack of available effective treatments for brain metastasis from BC are concerns [74,75]. Brain metastasis is diagnosed in about 25% of advanced BC patients and greatly diminishes the quality of life and OS. There is a higher chance of HER2-positive and TNBC developing brain metastasis compared to luminal breast cancer [2,3].

**Figure 4:**
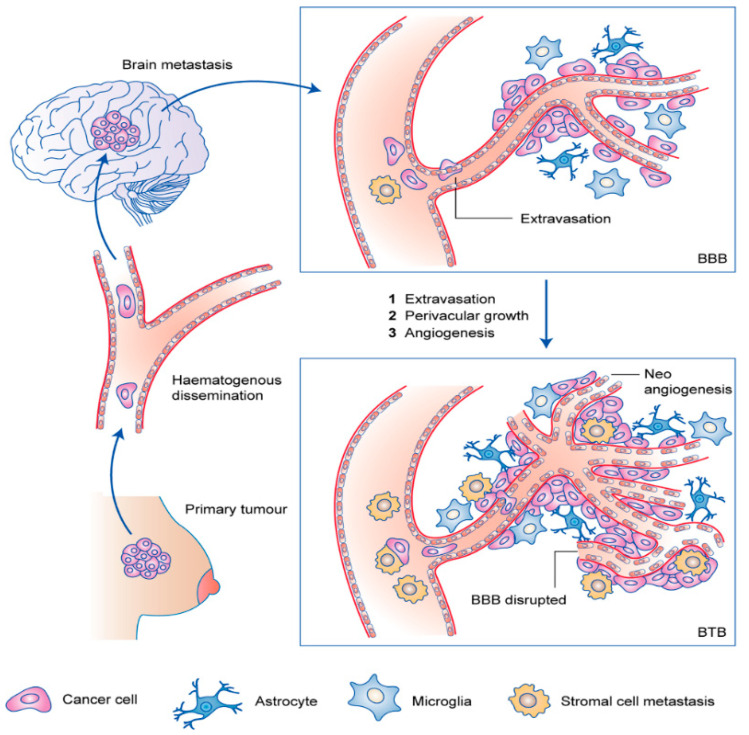
Mechanisms associated with the formation of hematogenous metastasis to the brain [80,81].

In contrast to other metastatic sites, BC brain metastases demonstrate a prolonged latency following the initial diagnosis of BC, due to the presence of the blood–brain barrier (BBB). The BBB is a highly specialized structure that consists of endothelial cells (ECs), tight junctions (TJs), astrocytes, pericytes, microglia, basement membranes, transporters, and enzymes. The BBB controls the permeability of the brain to macromolecules and is implicated in transmitting signals and upholding central nervous system homeostasis [76,77]. Hence, discovery of the biological pathways and regulatory molecules involved in the movement of biomolecules across the BBB is fundamental for BC brain metastasis prevention. Most cancer cells, including BC, utilize a paracellular mode of entry through BBB, whereby BCCs traverse through ECs by disrupting the intercellular junctions. Elongated metastatic cells round up and form cytoplasmic protrusions to expand the surrounding vascular wall, before or during the process of extravasation, with this process happening exclusively in capillaries and post-capillary venules that lack smooth muscle cells [76,78]. Intriguingly, the disruption to EC junctions seems to be repaired after paracellular extravasation, without substantial damage to the BBB [27]. As BC cells gather along the brain ECs, the previously intact BBB transforms into a permeable blood–tumor barrier (BTB) through vascular remodeling of pre-existing brain vessels, along with downregulation of the basement collagen membrane part IV and laminin α2 [27,79]. Subsequently, a change in pericyte phenotype involving the altered expression of desmin and CD13 affects the permeability of the barrier, as seen in BC brain metastasis [79]. These changes allow metastatic BC cells to easily cross the BBB. The steps involved in brain metastasis of breast cancer are illustrated in Figure 4 [80,81].

**Figure 5:**
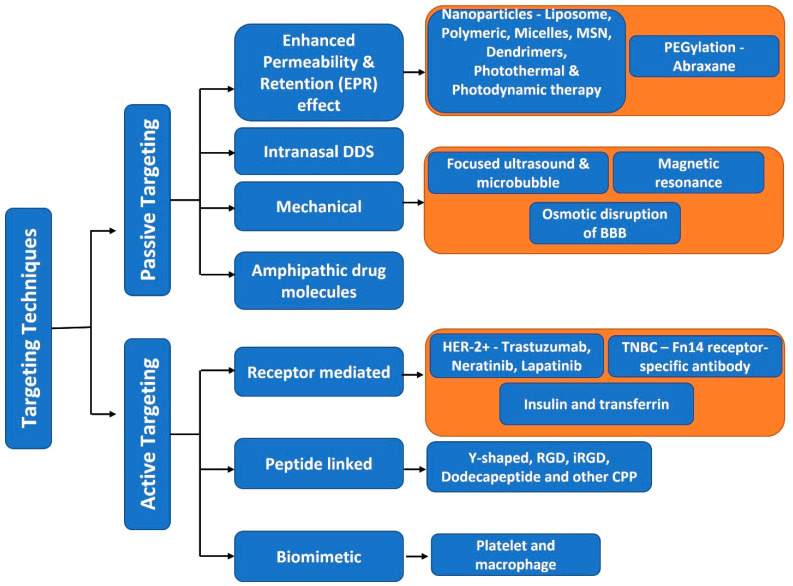
Different strategies utilized for the active and passive targeting against brain metastases of BC to overcome the hurdles associated with the BBB.

Treatment for brain metastases of BC consists of corticosteroids, along with surgical resection, whole-brain radiation therapy, stereotactic radiosurgery, and/or chemotherapy. Corticosteroids are employed to relieve the symptoms by reducing cerebral edema surrounding brain metastases [82]. Whole-brain radiation therapy (WBRT) is regarded as the most general choice of treatment for patients who present with multiple brain metastases [83]. Surgical resection of brain metastases allows for pathological diagnosis of the intracranial disease. Generally, chemotherapy is not considered a useful strategy in the management of brain metastases, as the tight junctions of the BBB impede the entry of most chemotherapeutic agents into the CNS [79,84]. Therefore, the vast majority of systemic chemotherapeutic agents are too large to cross the blood–brain barrier and result in inadequate drug delivery. Thus, nanotherapeutic approaches are considered a promising venue regarding the delivery of therapeutic agents as a treatment for BC brain metastasis. Key features of NPs that offer increased transport across the BBB and/or blood–brain–tumor barrier (BBTB) are depicted in Figure 5.

Recent clinical trials have elucidated the promise of targeting agents for HER2-positive BC brain metastasis treatment. Recently, neratinib, an HER2-targeted tyrosine kinase inhibitor in combination with capecitabine showed improved efficacy in treating HER2- positive BC brain metastasis [85]. The small-molecule inhibitor of EGFR and HER2, lapatinib, was found to cross the BBB [45]; however, it offered restricted activity when used as a single agent for HER2-positive BC brain metastasis. A combination of lapatinib with capecitabine has shown promising efficacy in BC brain metastasis models [86,87]. Trastuzumab–emtansine (T-DM1), an antibody–drug conjugate, is an approved second-line treatment for metastatic HER2-positive tumors following trastuzumab treatment alone [88]. Several other targeted therapies for BC brain metastasis are under clinical evaluation; for example, GDC-0084, a PI3K/Akt/mTOR pathway inhibitor, is being used in combination with trastuzumab for HER2-positive BC brain metastasis (ClinicalTrials.gov identifier: NCT03765983) [89]. Similarly, everolimus, a brain-permeable mTOR inhibitor [53], combined with trastuzumab and vinorelbine is being evaluated for HER2-positive BC brain metastasis treatment [90]. Ketoconazole is being used for treating patients with recurrent glioma or BC brain metastasis [83]. The antifungal drug ketoconazole blocks the effect of tGLI1, a protein involved in upregulation of the cancer stem-cell subpopulation and in the activation of astrocytes in glioblastomas [91].

In addition to the different inhibitors of cell signaling pathways that can cross the BBB, loading of drugs in nanosized particles and their functionalization with antibodies and peptide fragments are well-documented nanotherapeutic approaches for the treatment of brain metastatic BC. Active targeting of nanoparticles across the BBB can also be achieved using stimulus-responsive nanocarriers such as light- and magnetic-responsive nanoparticles. Some examples of these nanoparticles and their therapeutic efficacies in brain metastatic BC are provided here.

### 4.1. Liposome-Based Delivery Carriers

Liposomes are versatile and robust delivery systems with the ability to cross the BBB [92]. The efficacy and potency of liposomal DDSs for BC brain metastasis were assessed in several studies. ‘Y-shaped’ peptide pVAP-decorated platelet hybrid liposomes loaded with cabazitaxel nanocrystals were developed for BC brain metastasis treatment [93]. ‘Y-shaped’ peptides were designed to specifically target the glucose-regulated protein 78 (GRP78), a specific cell-surface marker present on cancer, neovascular, and ECs, which displayed potential BTB penetration ability [94]. The hybrid liposomes were made by incorporating egg yolk lecithin, cholesterol, DSPE–PEG3400–pVAP, and activated platelet membranes through the thin-film hydration (TFH) method. The pVAP peptide-decorated platelet membrane hybrid liposome retained native affinity toward circulating tumor cells (CTCs) via activated platelet membranes. In vivo and in vitro results showed that liposome-based DDSs have the ability to penetrate the BBB/BTB and accumulate in orthotopic breast tumors (4T1) and brain metastatic lesions. The peptide-functionalized liposomal DDSs also exhibited excellent antitumor efficacy and inhibition of brain metastatic TNBC when compared with the free drug [94].

Du and his team developed a liposome-based DDS for simultaneous delivery of chemotherapeutics and siRNA in drug-resistant cancer cell lines. Linear dodecapeptide peptide (BRBP1)-functionalized cholesterol- and distearoylphosphatidylcholine (DSPC)-based liposomes were developed to deliver paclitaxel and siRNA specific to the drug-resistant gene twinfilin-1 (TWF1) in TNBC tumor xenografts and in a brain metastatic mouse model (231-BR) [95]. Similarly, irinotecan delivery by a liposome-based DDS provided a therapeutic strategy for brain metastatic TNBC treatment. Fluorescence labeling of the DDS revealed that liposome-based carriers are able to cross the BBB and BTB, and gather in brain metastatic sites [96]. A phase I/II clinical study proved the successful use of PEGylated liposome-incorporated doxorubicin (Stealth^®^ liposomal doxorubicin (Caelyx^®^)) for both glioblastoma and BC brain metastasis. Radiolabeled Caelyx^®^ resulted in the accumulation of liposome inside the metastatic site in patient with BC-BM [97]. Another phase II clinical trial study indicated the successful use of temozolomide (200 mg/m^2^, days 1–5) with doxorubicin-loaded PEGylated liposomes (35 mg/m^2^, 28 days cycle), as a treatment regimen for BM. According to the survival rates, the test group showed a 50% 5 year median survival rate compared to the monotherapy [98]. Wu and the team tested doxorubicin-loaded PEGylated liposomes with focused ultrasound (FU) hyperthermia for 4T1 cancer-bearing mouse model treatment. The system showed enhanced doxorubicin accumulation within brain tumors compared to the non-FU control mice [99]. A phase I clinical study showed that liposomal cyterbine was a good choice for the treatment of newly diagnosed leptomeningeal metastasis for breast cancer. The median OS was established as 4.0 months (95% CI: 2.2–6.3) in the controls, compared to 7.3 months (95% CI: 3.9–9.6) for the experimental group [100].

### 4.2. Photothermal and Photodynamic Therapies

Photothermal and photodynamic therapies are devised to specifically destroy the cancerous tissue via the conversion of light energy into thermal and oxidative stress, respectively [101]. Selective photothermal absorbers can target difficult-to-treat tumors with marginal invasiveness [102]. However, the benefits of photothermal therapy (PTT) are critically limited by the heat tolerance of cancer cells following the overexpression of heat-shock proteins (HSPs) and the formation of stress granules that control protein expression and cell viability under different stress conditions [103]. PEG–lauric acid-conjugated pH-responsive peptide (PEG-pHLIP)-embellished hollow copper sulfide nanoparticles (HCuS NPs) encapsulating stress granules inhibitors (ISRIB) were developed to construct a pH-driven and near-infrared (NIR)-responsive controlled drug delivery system (IL@H-PP) for both primary and brain metastasis breast cancer treatment [104]. The stimulus-responsive nanoparticles specifically targeted marginally acidic tumor sites. In conjunction with NIR irradiation, the controlled release of ISRIB could efficiently inhibit the formation of PTT-induced stress granules to sensitize tumor cells to PTT, thus improving the antitumor effects, and subsequently inducing potent immunogenic cell death [104]. In addition, IL@H-PP could advance the production of reactive oxygen species (ROS) from tumor-associated macrophages, repolarizing them toward the M1 phenotype and remodeling the immunosuppressive microenvironment [104]. In vitro and in vivo investigations revealed the benefit of PTT combined with SG inhibitors, providing a new paradigm for BC and BC brain metastasis [104].

### 4.3. Polymeric Nanoparticles

A recent study suggested the use of Fn14-specific antibody-conjugated PLGA–PEG nanoparticles as an alternative delivery method for brain metastatic TNBC treatment [105]. Fn14 receptors are overexpressed on the brain metastatic TNBC cell surface, and a Fn14-targeted DDS has the potential to decrease nonspecific toxicity; this approach was termed as DART (decreased nonspecific adhesivity and receptor targeting). Fn14-targeted paclitaxel-loaded PLGA–PEG nanoparticles (NPs) were found to be more efficacious in a mouse model of brain metastatic TNBC than Abraxane, an FDA-approved paclitaxel nano-formulation for TNBC treatment [105]. The results showed that both Abraxane and Fn14-targeted nanoparticles exhibited similar accumulation in brains harboring TNBC tumors; however, Fn14-targeted DARTs exhibited a significant and precise association with Fn14-positive TNBC cells when compared to nontargeted NPs or Abraxane [105].

A terpolymer–lipid hybrid nanoparticle (TPLN) construct was developed with several targeting moieties to initially undergo synchronized BBB crossing and then aggressively target TNBC cells and tumor-associated macrophages (TAMs) in microlesions of brain metastases [106]. In vitro and in vivo investigations demonstrated that covalently bound polysorbate80 in the terpolymer facilitates low-density lipoprotein-enabled receptor-mediated BBB crossing and TAM targetability of the TPLN [106]. Conjugation of cyclic internalizing peptide (iRGD) enhanced cellular uptake, cytotoxicity, and drug delivery to the brain metastatic region of integrin-overexpressing TNBC cells [106]. iRGD-TPLN coloaded with doxorubicin (DOX) and mitomycin C (MMC) (iRGD-DMTPLN) exhibited higher efficacy in reducing metastatic burden and TAM depletion than nontargeted DMTPLN or a free DOX/MMC combined treatment [106]. iRGD-DMTPLN treatment reduced the metastatic burden sixfold and 19-fold respectively, and increased the host median survival time 1.3-fold and 1.6-fold, respectively, when compared to DMTPLN and free DOX/MMC treatment groups [106]. These outcomes indicate the potential of iRGD-DMTPLN as a multitargeted drug delivery carrier for the treatment of integrin-overexpressing brain metastases of TNBC [106].

Wyatt and Davis compared the uptake and antitumor efficacies of transferrin receptor (TfR)-targeted, camptothecin-conjugated polymeric nanoparticles in three BC brain metastasis models [107]. Breast metastatic brain tumors were established in murine models by intracranial (IC), intracardiac (ICD), and intravenous (IV) injections of human BC cells to develop tumors in mice that eventually metastasize to the brain [107]. The study showed that the approach used to establish tumors and their metastases in brain significantly impacted the BBB/BTB penetration capability of therapeutics [107]. Free drug accumulated in brain tumors formed post-IC and -ICD injections of BCCs and delayed the tumor growth, while nontargeted nanoparticles showed uptake and inhibition only in IC-established metastases [107]. TfR-targeted nanoparticles accumulated and substantially delayed the growth in all three models, suggesting that the IV model maintains a more intact BBB and BTB than the other studied models [107].

Hyaluronic acid is one of major substances found in the extracellular matrix; it helps maintain cell-to-cell adhesion and ultimately forms the regular tissue structure. Hyaluronidase is an endoglycosidase that breaks down hyaluronic acid and helps to separate individual cells or cell clusters. Hyaluronidase-catalyzed cell dissociation then enables the migration of BC cells to other distinct organs and initiate metastasis. Hyaluronidase is overexpressed in metastatic tissues when contrasted to normal cells, and their treatment with hyaluronic acid (HA) suppresses tumor growth [108]. Therefore, hyaluronic acid-conjugated drugs are thought to be cleaved and activated in the cytoplasm of metastatic BC. A group of researchers developed hyaluronic acid–doxorubicin (hDOX) conjugates and loaded them into hyaluronic acid comodified poly(lactic-co-glycolic acid)-poly(ε-carbobenzoxy-_L_-lysine) nanoparticles [109]. The system was further modified using transcytosis-targeting peptide (TTP), where hDOX exhibited enzyme-recovered DNA insertion and selective cytotoxicity to brain metastatic breast cancer cells rather than astrocytes, as well as effective loading into dual-targeting NPs [109]. hDOX@NPs demonstrated the dual-targeting ability of BBB and metastatic BC and substantially extended the median survival time of mice with intracranial metastatic BC [109].

### 4.4. Other Drug Delivery Carriers

The cellular Trojan horse concept uses endogenous cells as payload carriers, which are designed to address the drawbacks that traditional nanotherapeutics encounter in a heterogenic tumor microenvironment [110,111]. Macrophages and monocytes are key examples of endogenous cells that can cross the BBB and reach brain metastatic site easily [111]. A combination of nanotherapeutics with endogenous cells may provide an alternative strategy to treat metastatic brain cancer. For example, blood monocytes loaded with a gold–silica nanoshell were developed to deliver therapeutic agents in brain metastatic regions [111]. The nanotherapeutic-activated macrophages were able to cross the BBB, as well as envelope the metastatic cells, allowing the delivery of the payload in the vicinity of metastatic cells [112]. This type of delivery system takes advantage of the morphological features of macrophages to enter the metastatic lesions with the help of chemoattractants and does not require the enhanced permeability and retention (EPR) effect or diffusion of the nanovector [112].

Whole-brain radiotherapy (WBRT) is an X-ray driven radiotherapy technique which is used as a first-line therapeutic option for BC brain metastasis [79]. The small molecules that can be activated in the presence of X-rays and generate free radicals to destroy tumor cells can enhance the efficacy of conventional WBRT [84]. Iodine NPs (INPs) have the ability to absorb X-rays during WBRT, creating free radicals and efficiently improving local radiotherapy at the tumor site. The effectiveness of INP was tested using a human triple-negative BC (TNBC, MDA-MB-231 cells) orthotopic model in athymic nude mouse brains [113]. A well-tolerated and nontoxic IV dose (7 g/kg) of the INPs showed the presence of an iodinated rim around the tumor tissue, and the average uptake of iodine by weight was discovered to be 2.9% [113]. The calculated dose enhancement factor for this formulation was approximately 5.5, which is the maximum ever reported for any radiation-enhancing agent [113]. Using RT alone (15 Gy, single dose), all mice expired by 72 days; INP pretreatment led to long-term remissions, with 40% of animals surviving for 150 days, whereas 30% survived for more than 280 days [113].

A micellar drug delivery system loaded with lapatinib (LPTN) and paclitaxel (PTX) was developed as a combined therapy approach for brain metastases [114]. The findings showed that the micelles modified with Angiopep-2 had high loading efficiency of lapatinib and paclitaxel (Ang-MIC-PTX/LP) [114]. Furthemore, Ang-MIC-PTX/LP could also be transported across in vitro BBB model and accumulate in BC cells. Following intravenous injection, Ang-MIC substantially accumulated in the brain metastatic sites and extended the lifespan of brain metastatic mouse models [114].

Trastuzumab-conjugated MSN nanoparticles were utilized for the co-delivery of docetaxel and siRNA against HER2 (siHER2) in a drug-resistant orthotopic HER2^+^ invasive ductal carcinoma model [115]. The targeted nano-constructs inhibited tumor growth more efficiently than the trastuzumab and docetaxel combination [115]. When combined with microbubble-assisted focused ultrasound, which transiently disrupts the BBB, the nano-constructs were found to inhibit the growth of trastuzumab-resistant HER2^+^ breast tumors inhabiting mouse brains. The nano-constructs also showed a favorable safety profile in vitro and in mice models [115].

## 5. Lung Metastasis of Breast Cancer

The incidence of lung metastasis for breast cancer is reported to be 40% and 20% in TNBC and non-TNBC patients, respectively [2]. Gene expression analysis showed that, in comparison to bone relapse, lung relapse was more abundant in patients diagnosed with the luminal B and basal subtypes of BC [116]. Remarkably, the absence of lung relapse was seen in the luminal A subtype, while brain metastasis was primarily found in patients with basal-like BC and HER2^+^ BC [8]. The survival data of patients with lung metastasis revealed that hormone receptor-positive BCs showed the ideal clinical outcome, while HER2^+^ cancers and TNBC had the worst prognosis [116]. The HER2^+^ subtype was also found to display a higher risk of developing liver metastasis [116].

Up-to-date therapeutic options mainly consist of chemotherapy, radiotherapy, and/or surgical resection; however, their long-term influence on survival (particularly when multiple pulmonary metastases are found) is still bleak and not effective enough to prevent a relapse [117].

**Figure 6:**
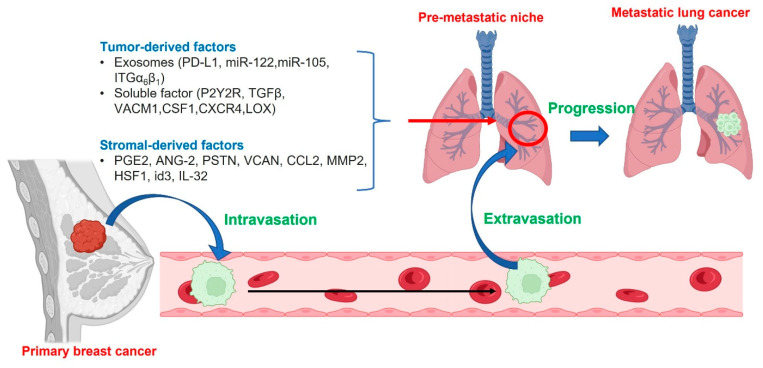
Schematic displaying the mechanistic steps associated with lung metastasis of BC.

For lung metastasis, there is a rate-limiting step that involves the penetration of BC cells through the lung vasculature [118]. However, CTC may come into contact with as much as 100 m^2^ of the vascular bed in lung through the circulation, and most of these microcapillaries are smaller than CTC. Therefore, there is a high chance of breast cancer cell arrest in these capillary beds, along with subsequent extravasation into the lung tissue [119]. However, survival of extravasated tumor cells and production of micro-metastasis within the lung tissue is quite rare due to innate immune responses. In order to surpass this hurdle, there are several proposed mechanisms. The first step is producing a favorable microenvironment within the lung tissue by the primary cancer cells called premetastatic niches. The process is started by the secretion of tumor-derived factors and is subsequently accelerated by factors secreted by stromal cells [120]. Furthermore, ANGPTL4, which is secreted by the primary tumor, has the ability to disrupt lung vascular endothelial lining and induces hyperpermeability of the capillaries, leading to the establishment of pulmonary metastases [121]. Disseminated cancer cells that enter this fertile metastatic niche may start their growth to form micro-metastases or remain in a dormant state until the circumstances at the secondary site can support tumor outgrowth [122,123]. The main steps of lung metastasis are described further in Figure 6.

The treatment of BC lung metastasis is mainly dependent on the type of cancer and its metastatic profile. The treatment regime that has been used for primary BC can also be used for BC lung metastasis. Hormonal therapy can be used for hormone receptor-positive cancers, while immunotherapy can be used for HER2^+^ BC. Chemotherapy is the only option for TNBC. Radiation therapy can help destroy cancer cells in a localized area, and stereotactic radiotherapy can also be used. Surgery is not a good option since most lung metastases are not localized tumors that can be removed by surgery [124]. Failure to deliver adequate amounts of drugs to the lung metastatic site is the main drawback of current chemotherapy treatments. Increasing the chemotherapeutic dose to achieve a better therapeutic index is known to produce severe toxicity to normal organs [125]. The lack of selectivity of chemotherapeutics is the main reason for the inadequate drug accumulation within the lung metastatic site [126]. Hence, a biomimetic approach becomes the most dynamic method to deliver therapeutic agents directly to the tumor sites, representing an emerging therapeutic for lung metastasis [127]. 

### 5.1. Biomimetic Delivery Systems

Biomimetic delivery systems comprise therapeutic agents or nanomaterials that are camouflaged with biological substances such as tumor specific host cells or cellular membranes. Examples of specific host cells include macrophages, T cells, mesenchymal stem cells, and red blood cells; however, macrophages are the most popular option due to their ability to penetrate the tumor site and accumulate inside the tumor without any difficulties [110,111]. The in vitro grown mouse macrophage-like cell line (RAW264.7) has been widely used for biomimetic drug delivery applications. Doxorubicin-loaded RAW264.7 cells were successfully tested in mouse 4T1 breast cancer cells as a biomimetic drug delivery system [128]. A therapeutically significant amount of DOX was loaded into the RAW264.7 cells using a simple incubation method without substantially affecting the viability of the cells [128]. The in vivo evaluation of DOX-loaded macrophages in 4T1 bearing mouse model indicated lifespan prolongation, promising anticancer efficacy in terms of tumor suppression, and metastatic inhibition, along with reduced toxicity [128].

A similar approach explored the potential of macrophage membrane (M)-camouflaged quercetin (QE)-loaded hollow bismuth selenide nanoparticles (M@BS-QE NPs) for hyperthermia-triggered drug release [129]. The NPs possessed extended circulation life, enhanced and accelerated tumoritropic accumulation, due to the immune-evading capacity, C–C chemokine ligand 2 (CCL2)-mediated recruitment properties of the macrophage membrane, and active targeting ability [129]. The ensuing QE release under near-infrared (NIR) laser irradiation specifically sensitized cancer cells to photothermal therapy (PTT) by exhausting heat-shock protein 70, resulting in a yielded cascaded synergistic effect of interest. M@BS-QE downregulated p-Akt and matrix metalloproteinase-9, which resulted in the degradation of the ECM and promoted the invasion and metastasis of tumors [129]. 

Cao and his team developed RAW 264.7 macrophage membrane-coated liposomes loaded with anticancer drug emtansine for BC lung metastasis treatment [130]. pH-sensitive emtansine-loaded liposomes were arranged by a thin-film hydration technique using distearoyl-phosphatidylethanolamine–poly(ethylene glycol) (DSPE–PEG) and 1,2-dioleoyl-sn-glycero-3-phoshoethanolamine (DOPE), coated with isolated macrophage membranes (MELs) [130]. MELs could efficiently target lung metastases foci, and their targeting ability was compromised when α4β1 integrin markers were blocked [130]. This is because macrophages aggressively bind metastatic cancer cells via α4β1 integrin–VCAM-1 interactions to prime the formation of lung metastatic lesions. Subsequently, the specific targeting to the lung metastases could be due to the biomimetic active targeting properties of the α4β1 integrin markers in macrophage membrane [130]. 

Another study showed the use of macrophages and 4T1 hybrid membrane-coated doxorubicin (Dox)-loaded poly(lactic-co-glycolic acid) (PLGA) NPs (DPLGA@[RAW-4T1] NPs) to treat lung metastases initiated from BC [131]. This investigation indicated that combining NPs with a hybrid membrane derived from macrophages and cancer cells has several advantages, including the propensity to accumulate at the inflammatory site and the ability to target specific metastatic regions, demonstrating homogenous tumor targeting abilities in vitro, and markedly enhancing the multitargeting capability in a lung metastatic model in vivo [131]. DPLGA@[RAW-4T1] NPs showed excellent chemotherapeutic capability with approximately 89% anti-metastasis efficacy due to the multitargeting ability of the nanoparticles [131]. Similarly, Sun et al. used 4T1 cell membrane-coated doxorubicin (DOX)-loaded gold nanocages (AuNs) for specific targeting of the homotypic tumor cells with hyperthermia-triggered drug release under NIR laser irradiation [132]. Macrophage membrane-coated, pH-sensitive doxorubicin prodrug-loaded liposomes were developed by Li et al., and their antitumor effects were investigated on BC lung metastasis. pH-sensitive liposomes comprised DNA tetrahedron dendrimers to intercalate the prodrug and contained a pH-sensitive linkage that was disintegrated under acidic conditions to trigger doxorubicin release in pathological conditions [133]. Red blood cell (RBC)-mimetic nanoparticles composed of a paclitaxel (PTX)-loaded polymeric core and a hydrophilic RBC vesicle shell showed a long circulation time with a half-life of (t_1/2_) 32.8 h, ~6-fold higher compared to bare NP [134]. In addition, the data demonstrated that the tumor penetration capacity of the RBC-mimetic NPs in the 4T1 cancer model was significantly improved by their co-administration with a tumor-penetrating peptide iRGD; the complex was shown to reduce over 90% of the tumor growth and suppress 95% of the lung metastasis [134].

### 5.2. Multifunctional Polymeric Nanoparticles

Polymeric nanoparticles including polyethylene glycol (PEG), polyethyleneimine, poly(*N*-(2-hydroxypropyl)methacrylamide) (PHPMA), and polyvinyl methyl ether (PVM) play a vital role as drug delivery systems. Dan and coworkers developed host–guest dual-polymer nano-systems to deliver succinobucol, a novel, effective, and selective inhibitor of VCAM-1, which is considered a promising candidate for reducing lung metastasis [135]. NPs composed of a β-cyclodextrin-based host polymer of N,N-diisopropylethylenediamine (βCD–DPA) and the guest polymer of adamantyl end-capped methoxy poly(ethylene glycol) (mPEG-Ad) exhibited specific and rapid drug release in response to intracellular acidic pH by breaking the βCD–DPA linkage [135]. In another study, PEGylated paclitaxel nanocrystals (PEG–PTX NCs) developed by Zhang et al. showed significantly better tumor inhibition in a 4T1 xenograft mice model. Furthermore, PEG–PTX NCs showed higher stability than PTX NCs during storage and under physiological conditions [136]. A similar study published in 2015 investigated the use of triblock copolymer poloxamer (P188)-based NPs to improve the bioavailability of inherently insoluble succinobucol and successfully delivered the drug to the target site [137].

Nanoparticles comprising a pH-sensitive core, a cationic shell, and a matrix metalloproteinase (MMP)-cleavable polyethylene glycol (PEG) corona conjugated through a peptide linker were used for dual delivery of paclitaxel and siRNA in a pulmonary metastatic BC model [138]. Degradation of the MMP-specific linker removed the PEG layer at the tumor site, yielding smaller particles size of higher net positive charges, and leading to an effective cellular uptake in tumor cells and higher intra-tumor accumulation of cargo in the 4T1 tumor-bearing mice models when compared to the nanoparticles with a fixed PEG layer [138]. Furthermore, acid-triggered drug release in endo/lysosomes was accomplished through the pH-sensitive core. As a result, the MMP/pH dual-sensitive nanoparticles significantly inhibited tumor growth and pulmonary metastasis [138]. 

The pH-sensitive amphiphilic polymer, poly [(1,4-butanediol)-diacrylate-β-*N,N*-diisopropylethylenediamine]-polyethyleneimine (BDP), was produced to deliver PTX and Akt-specific siRNA for PTX-resistant BC lung metastasis treatment [139]. Silencing of Akt expression interferes with the PI3K/AKT/mTOR signaling pathway, abrogating the drug resistance in cancer cells [139]. The nano-system developed showed pH-mediated drug release in the endo/lysosome-mediated acidic environment [139]. In 4T1 cells, downregulation of Akt and P-glycoprotein (P-gp) upregulated the expression of Caspase-3 [139]. The downregulated P-gp also inhibited the efflux of PTX, thereby amassing its intracellular concentration. In 4T1 tumor-bearing mice, co-delivery of PTX and siAkt achieved a tumor inhibition rate of 94.1% and suppressed 96.8% of lung metastasis [139]. 

Innovative, redox-responsive, chondroitin sulfate-based nanoparticles that could concurrently deliver quercetin (chemosensitizer), chlorin e6 (photosensitizer), and PTX (chemotherapeutic agent) were developed as chemo-photodynamic therapy for affecting the MDR and lung metastasis of BC [140]. In vitro studies showed that nanoparticles downregulated the expression of P-gp in MCF-7/ADR cells [140]. In addition, NIR laser irradiation of nanoparticles generated cellular ROS, resulting in mitochondrial membrane potential loss, and facilitated the lysosomal escape of drugs [140].

Another study used polymeric nanoparticles of (poly-*N*-(2-hydroxypropyl) methacrylamide (pHPMA) coated with wheat germ agglutinin-modified phosphatidylcholine lipid–polymer hybrid nanoparticles to deliver natural anticancer plant products silibinin and cryptotanshinone [141]. The inadequate aqueous solubility, membrane permeability, and reduced oral bioavailability of silibinin and cryptotanshinone was improved by the nanoparticle formulations [141]. The formulated NPs downregulated tumor microenvironment biomarkers such as CD31, TGF-β1, and MMP-9 that promote metastasis [141]. A nanodiamond (ND)-mediated doxorubicin (DOX)-loaded delivery system was developed to effectively reduce the lung metastasis of BC [142]. Here, DOX was noncovalently bound to NDs through physical adsorption in aqueous solution and was then coated with lipid-modified PEG conjugates (DSPE-PEG 2K). DSPE-PEG 2K-coated NDX (DNX) displayed high drug loading and excellent ability to deliver DOX to the tumor cell nuclei, thereby significantly enhancing cytotoxicity and inducing cell apoptosis [142].

Xu and the team designed a multifunctional nano-micellar carrier, established with conjugation of an amphiphilic copolymer POEG–VBC backbone functionalized with creatine to co-deliver bioengineered miRNA prodrug (tRNA-mir-34a) and DOX [143]. miRNA-34a is often downregulated in TNBC; it is responsible for induced cell apoptosis and inhibits cell proliferation [143]. Therefore, DOX + tRNA-mir-34a-carrying nanoparticles exhibited potent synergistic antitumor and antimetastatic activity in vitro and in vivo [143]. Further investigations suggested that the enhanced immune response produced by DOX was responsible for the overall antitumor efficacy [143]. 

In another approach, the therapeutic efficacy of PEGylated doxorubicin-loaded liposomes (DOX-L) was enhanced by reducing the action of cancer-associated fibroblasts (CAFs) [144]. CAFs are considered an accessory to tumor growth and promote survival, growth, and metastasis of cancer cells [144]. A losartan-loaded peptide-based hydrogel depot was prepared to deter the activity of CAFs, by prolonging the localized drug exposure [144]. After intratumoral injection, the hydrogels preserved in the tumor tissue for more than 9 days substantially inhibited CAFs and reduced collagen synthesis in orthotropic 4T1 tumors, and enhanced the efficacy of Dox-L in inhibiting the tumor growth (64% vs. Dox-L alone) and lung metastasis (80% vs. Dox-L alone) [144].

Due to their small size, nanoparticles possess superior capability to extravasate the tumor tissue through the EPR effect. Gold NPs are among the biocompatible NPs being studied for cancer treatment due to their unique photodynamic properties. For example, CD44-targeted, hyaluronic acid (HA) and bovine serum albumin-protected gold nanoclusters (AuNC@BSA) were developed to construct a size-reducible theranostic nanoplatform [145]. The nanoparticles were further loaded with PTX in order to achieve chemo-photothermal dual therapy [145]. The nano-system showed a size reduction in the presence of hyaluronidase and showed high accumulation in BC lung metastasis [145]. A similar study published in 2019 described the application of hyaluronidase-responsive size-reducible biomimetic gold nanocluster (mCAuNCs@HA) in improving the blood circulation, tumor penetration, and retention capability [146]. The nanoparticles were loaded with photosensitizer pheophorbide A and ROS-responsive PTX dimer prodrug (PXTK) to enable on-demand drug release and were embellished with PD-L1 peptide (dPPA) to alleviate the immunosuppressive environment of the tumor [146]. The combination therapy activated CD4^+^, CD8^+^ T cells, and NK cells, enhanced the secretion of proinflammatory cytokines (TNF-α and IL-12), resulting in a tumor inhibition rate of 84.2%, and no metastasis was recorded [146]. Li et al. developed a multifunctional phototheranostic nanoprobe consisting of chlorin e6 (Ce6)-conjugated, polydopamine (PDA)-coated gold nanostars (AuNSs) for theranostic applications. The system was demonstrated to contain concurrent photoacoustic (PA) imaging, photothermal therapy (PTT), and photodynamic therapy (PDT) abilities [147]. Another study showed the use of doxorubicin-loaded, iron oxide nanoparticle-incorporated, magnetic single-walled carbon nanotubes (SWCNTs) for lung metastasis in the presence of a high-energy multipole magnet applied over the tumor. The conjugation of monoclonal anti-CD105 to SWCNT enabled tumor-specific accumulation. Therefore, magnetic- and immunology-guided nanoparticles showed significant accumulation inside the tumor under MRI [148].

Fullerenol is a derivative of fullerene with an additional binding site created with a hydroxyl group that can enhance the drug loading efficacy. Therefore, fullerenol nanoparticles can be used as a carrier system to deliver bioactive molecules to the tumor site [149]. In addition, several studies showed the use of a variety of DDSs that can be used as selective and targeted delivery of materials to BC lung metastatic sites. Regenerated silk fibroin-based nanoparticles [150], marine sulfated polysaccharide-derived NPs [151], and layered double hydroxide (LDH) nanoparticles are some examples.

Given the above-noted success of nano-therapeutics in laboratory settings, their applications in clinical settings are gaining momentum. Following the approval of the first nanomedicine Doxil^®^ by the Food and Drug Administration (FDA) for the treatment of various cancers, including metastatic breast cancer, numerous nanobased therapies for cancer treatment have emerged globally. These drug delivery systems (DDSs) can be broadly categorized into liposomal formulations (such as Doxil^TM^, Caelyx^TM^, Myocet^TM^, DaunoXome^TM^, Mepact^TM^, and Lipusu^TM^), protein–drug conjugates (e.g., Abraxane^TM^ and Pazenir^TM^), and polymeric micelles (e.g., Genexol-PM) [152,153,154,155,156,157].

Liposomal anthracycline formulations, such as Doxil/Caelyx, Myocet, and DaunoXome, have been approved for second-line treatment and have shown promising efficacies as single agents and in combination with taxanes, cyclophosphamide, vinorelbine, and gemcitabine in patients with metastatic breast cancer. Doxil/Caelyx, in particular, is a PEGylated liposomal doxorubicin formulation of ~100 nm, where the liposome is coated with a PEG layer (known as Stealth™ technology, Alza Pharmaceuticals Inc., Mountain View, CA, USA) to protect it from phagocytes. Furthermore, PEGylated liposomal doxorubicin exhibits preferential uptake by tumor cells compared to normal cells in patients with breast and other solid cancers [152,153].

Abraxane, an albumin-bound nanoparticle of paclitaxel, has been approved by the FDA as a first-line therapy for metastatic breast cancer, as well as for the first-line treatment of advanced non-small-cell lung cancer and late-stage pancreatic cancer. In metastatic breast cancer patients, Abraxane has demonstrated superior efficacy compared to Taxol [156,157]. Genexol-PM is a lyophilized polymeric micellar formulation of PTX that enables higher drug delivery to tumor tissue while reducing vehicle-related toxicities compared to conventional paclitaxel formulations. Genexol-PM is prepared using a low-molecular-weight, biodegradable amphiphilic diblock copolymer known as mPEG-PDLLA (methoxy-polyethylene glycol-block-poly(D,L-lactide)) [152,155].

As indicated on the National Institutes of Health (NIH) website (https://clinicaltrials.gov/, (accessed on 15 May 2023)), there were more than 250 nanotechnology-based cancer treatments undergoing clinical trials in 2022. The clinical translation of nanomedicine entails a complex and time-consuming process. Compared to conventional formulations, nanomedicine faces greater challenges in designing drug delivery systems, establishing evaluation profiles, scaling up manufacturing techniques, and overcoming regulatory barriers. Although the number and formulation of approved nanodrug delivery systems for cancer treatment are still limited, the development of versatile nanomedicines for clinical use now extends beyond cytotoxic drugs to include therapeutic agents such as siRNA, miRNA, and kinase inhibitors [152,154]. Table 1 summarizes the key features of nanoparticles that are under laboratory and clinical investigations for metastatic BC treatment.

## 6. Conclusions

Metastatic BC is the most aggressive type of breast cancer with poor prognosis. Nanotherapeutics can be considered as the only hope to combat metastatic BC, since conventional therapies are associated with a number of drawbacks including low compliance, therapeutic failure, and affordability. These drawbacks can be successfully masked by combining conventional therapy with nanotechnology and producing novel formulations with the following properties: (1) alteration of the physicochemical properties of the payload to enhance bioavailability issues; (2) increase in the therapeutic index of drugs by a slow or sustained release platform; (3) augmentation of the metabolic stability and plasma circulation time of the payloads; (4) encouraged passive and active targeting through targeted DDSs using different targeting moieties; (5) facilitation of the triggered release of the payloads; (6) combination of multiple anticancer agents in a single DDS. Collectively, these features are all essential for the development of effective nanotherapeutic strategies. However, thus far, more attention has been focused on the targeting and synergistic effects of nanocarriers. 

HA and ανß3 integrin are the most common targets for BC bone metastasis. Therefore, bisphosphonate such as ALN and ZLA are the most effective targeting agents for BC bone metastasis treatment. In addition, as targeting agents, bisphosphonates are able to suppress the RANKL system, and then inhibit the osteoclast activation and bone resorption, resulting in enhanced patient compliance and quality of life. Developing targeting agents for brain metastasis is challenging due to the combined effect of the BBB and BTB. HER-2-targeted therapy can be used for brain metastasis because the majority of brain metastases are HER2-positive. Nanotherapeutics with the ability to create ROS in the presence of radiation have added advantages, showing promising results with whole-brain radiation therapy. Therefore, WBRT with nanotherapeutics has become a more reliable therapeutic system, and further research is needed in order to optimize this drug delivery system for clinical settings. Recent research has specifically focused on a Trojan horse approach where DDSs are camouflaged with biological membranes to precisely deliver payload in metastatic sites. This biomimetic approach is specifically common in brain and lung metastases due to the structural complexity of these metastatic lesions. In this regard, macrophage-, RBC-, and platelet-coated DDSs have shown promising targeting potential for metastatic BCs. RGD and iRGD peptides other types of targeting molecules that are heavily used in metastasis DDSs. 

Nonetheless, being in their early preclinical stages of progression, there are still challenges for the future translation of these technologies to the clinic. Some of these challenges comprise optimizing the fabrication process for upscaling to reach clinical translation, as well as designing and conducting studies that let us know about the fate of DDSs in vivo, their interactions with blood, healthy, and diseased tissues, and cells, and their accumulation in the intracellular compartments [158,159,160]. In addition, lessons from the previous development of nanomedicines for various diseases have shown that DDSs with more complex designs face additional challenges in their optimization and characterization that lead to lower reproducibility in the production processes [159,161]. To fully reveal the potential of nanotherapeutics for metastatic BC, it is imperative that we understand the properties of nanomaterials, as well as the pathology and physiology of the metastatic process itself. This will result in more in-depth investigations of the interaction between the DDS and the BC metastatic sites, thus optimizing the design of nanocarriers for metastatic BC treatments.

## Figures and Tables

**Table 1:** Summary of nanotherapeutics being studied for metastatic BC treatment.

Type of Nanoparticle	Composition	Special Features/Advantages	Targeting Agent	Payload	Ref
	**Nanotherapeutics for bone metastasis**				
Coordination polymer	Inner core—DSP and Zn Outer stealth—ALN PEG conjugate	Reduced toxicity of cisplatin Analgesic effect 4-fold drug accumulation in metastatic site compared to the normal bone	ALN	Cisplatin prodrug (DSP)	[58]
Polymeric	PLGA	Dual-targeted Prolonged median survival rate (>50 days), compared to the single targeted ALN (35 days) and FA (27 days)	ALN and FA	Paclitaxel (PTX)	[59]
Nanocluster	Human serum albumin	5.04-fold higher affinity compared to the nontargeted nanocluster	ALN	Doxorubicin (DOX)	[60]
Micelles	Oligosaccharide hyaluronan	Redox-sensitive Drug release dependent on glutathione	ALN	curcumin	[61]
Polymeric	PLGA	Dual delivery system 2-fold decrease in tumor volume after 20 days compared to curcumin	ALN	Curcumin and bortezomib	[62]
Micelles	PEG and polylysine (PEG-*b*-PLL)	Acid liable aryl boric ester linkage Reduced systemic toxicity and improved therapeutic effect (3-fold) compared to free drug	ALN	bortezomib	[54]
Lipid/oil	Triptolide	High loading efficacy and biphasic control Retention in the bones up to 8.1-fold versus free drug	ALN	PTX or Docetaxel (DOT)	[63]
Micelles	PEG, PGlu, and PPhA copolymer	Biodegradable Remodeling ability of the tumor–bone microenvironment	ALN	DOT	[64]
Polymeric	PEG–hyaluronic acid and poly(aspartic acid)	pH- and redox-sensitive Glutathione-, dithiothreitol-, and pH-dependent drug release	ALN	DOX	[65]
GO nanosheet	Graphene oxide	Nanosheet as DDS 55% more accumulation of NP in bone compared to the nonfunctionalized Np	ALN	DOX	[66]
Nanoparticle	Zinc phthalocyanine	Chemo-photodynamic therapy Tumor volume cut down by 85% compared to the PBS	ALN	Bortezomib	[67]
Polymeric	Amphiphilic deblock copolymers	Inhibition of osteoclast activation Ability to decrease tumor-bone lesion 3-fold and increase bone volume fraction 2.5-fold	ALN	GANT58	[68]
Polymeric	PEGylated polybutyl cyanoacrylate	Inhibition of FPP and acceleration of apoptosis 7- and 5.3-fold increases in IPP and ApppI production compared to ZLA	ZLA	DOT	[70]
MSN	Gold nanorod-loaded MSN	Photothermal therapy Inhibition of the formation of osteoclast-like cells and promotion of osteoblast differentiation	ZLA	-	[53]
Nanoparticle	Fe_3_O_4_ and ICG-entrapped PLGA	Photothermal therapy with magnetic NP Dual-targeting and double photothermal effect Accumulation inside the bone with the presence of magnetic field	ZLA	-	[71]
Nanoparticle	Metal–organic framework	Inhibition of osteoclast formation and induction of macrophage polarization Strong affinity to calcium phosphate	ZLA	Cytosine–phosphate–guanosine	[72]
Dendrimer	Poly(amidoamine)	pH-responsive dendrimer Inhibition of the progression of metastatic bone tumors and tumor-associated osteolysis	RGD and cRGD	Bortezomib	[57]
Micelles	Phospholipid/polysorbate 80	6.5-fold affinity for bone and less hepatotoxicity	αvβ3-targeted quinolone nonpeptide	DOT	[73]
	**Nanotherapeutics for brain metastasis**				
Liposome	Lecithin, cholesterol, DSPE–PEG3400–pVAP and platelet membrane	Platelet-hybrid liposome DDS Survival time (36.5 days) longer than compared to DOX-loaded plain liposomes (26.5 days)	‘Y’-shaped peptide	Cabazitaxel	[93]
Liposome	Cholesterol and DSPC	Dual delivery system Delivery of siRNA to knockdown TWF1 and enhance sensitivity to PTX Ability to cross BBB via BRBP1 modification	BRBP1 linear dodecapeptide	PTX and siRNA for TWF1	[95]
Liposome	DSPC, cholesterol, and PEG–DSPE	Depot for sustained release of irinotecan Prolonged plasma drug exposure Median survival of 50 days, compared to 37 days for pure drug	-	Irinotecan	[96]
Liposome	PEG (Stealth^®^)	Phase II clinical trial Complete response in 40% of treated patients	-	DOX (Caelyx^®^)	[96]
Liposome	PEG (Stealth^®^)	Phase II clinical trial Median overall survival of 10 months (95% CI: 6.3–13.7)	-	Temozolamide and DOX	[98]
Liposome	PEG, phospholipid, cholesterol	Tested with FU hyperthermia, significant tumor growth inhibition compared to the liposome alone	-	DOX	[99]
Liposome	DEPOSEIN^®^	Phase II clinical trial for leptomeningeal metastasis Median overall survival of 7.3 months compared to 4.0 months for control	-	DOX	[100]
Hallow nanoparticle	PEG–lauric acid conjugate copper sulfide	Photothermal therapy Promotion of ROS generation and macrophage repolarization	-	Stress granule inhibitor (ISRIB)	[104]
Polymeric	PLGA–PEG	Fn14-directed “DART” DDS More effective (7.8-fold) than Abraxane®	Fn14-specific antibody	PTX	[105]
Nanoparticle	Terpolymer and polysorbate80	Multitargeted DDS LDL receptor-mediated BBB crossing by polysorbate80	iRGD	DOX and mitomycin	[106]
Polymeric	Mucic acid polymer	Whole-brain therapeutic accumulation Transferrin receptor-mediated BBB crossing	Transferrin	Camptothecin	[107]
Polymeric	Poly(lactic-co-glycolic acid)–poly(ε-carbobenzoxy-L-lysine)	Dual-targeting DDS Selective cytotoxicity to cancer cells over astrocytes	Hyaluronic acid transcytosis-targeting peptide	DOX	[109]
Nanoparticle	Iodine	Effective boost of local radiotherapy 2-fold higher median survival compared to WBRT alone	-	-	[113]
Micelles	PEG and PLA	Dual DDS LDL receptor-mediated BBB crossing by Angiopep-2	Angiopep-2	PTX and Iapatinib	[114]
MSN	Silica	Effective for trastuzumab-resistant HER2+ brain tumor Coupled with microbubble-assisted focused ultrasound	Trastuzumab	PTX and siHER2	[115]
	**Nanotherapeutics for lung metastasis**				
Biomimetic intact cell	RAW264.7 cell	Use of drug-loaded intact cells Improved survival (80%) compared to drug only (30%) after 30 days	-	DOX	[128]
Biomimetic-Membrane	Macrophage membrane-camouflaged quercetin-loaded bismuth selenide nanoparticle	Hyperthermia-triggered drug release Downregulation of p-Akt and MMP-9 Depletion of heat-shock protein 70	-	Quercetin	[129]
Biomimetic membrane-coated liposome	RAW264.7 macrophage membrane, DSPE–PEG, and DOPE	pH-sensitive drug release 3-fold reduction in metastatic node compared to drug alone	-	Emtansine	[130]
Biomimetic membrane-coated polymeric NP	RAW264.7 and 4T1 membrane, and PLGA	Hybrid biomimetic membrane 88.9% antimetastatic efficacy compared to the control	-	DOX	[131]
Biomimetic membrane-coated nanocage	4T1 membrane and gold nanocage	Hyperthermia-triggered drug release Decrease in metastasis by 98.9% compared to the control	-	DOX	[132]
Biomimetic membrane-coated liposome	RAW264.7 membrane and DNA tetrahedron dendrimers	pH-sensitive drug release Decrease in metastasis by 78% compared to the control	-	DOX	[133]
Biomimetic membrane-coated polymeric NP	RBC membrane and PCL	Extended circulation time (5.8-fold) and antimetastatic (90%) ability compared to the polymeric NP	iRGD	PTX	[134]
Polymeric NP	βCD–DPA and mPEG-Ad	Host–guest dual-polymer DDS Inhibitory effects on cell migration and invasion activities, VCAM-1 expression	-	Succinobucol	[135]
Polymeric NP	Triblock copolymer poloxamer	Oral DDS 13-fold increased oral bioavailability of succinobucol	-	Succinobucol	[137]
Core–shell polymeric NP	PEI–PDHA core and PEG–PDHA shell	Dual delivery system Acid-triggered release of drug chemotherapeutic drugs in endo/lysosomes	-	PTX and Twist-targeting siRNA	[138]
Polymeric NP	BDP	pH-mediated intra-endo/lysosome drug release Downregulation of Akt and P-glycoprotein and upregulation of Caspase-3	-	PTX and Akt-specific siRNA	[139]
Polymeric NP	Chondroitin sulfate	Chemo-photodynamic therapy Downregulation of P-glycoprotein		Quercetin, chlorin e6, and PTX	[140]
Lipid-Polymeric NP	pHPMA and wheat germ agglutin-modified phosphatidylcholine	Facilitation of the delivery of water-insoluble drug Downregulation of CD31, TGF-β1, and MMP-9	-	Silibinin and cryptotanshinone	[141]
Nanodiamond	DSPE–PEG-coated nanodiamond	Nanodiamond-based DDS Focused delivery of DOX to nuclei	-	DOX	[142]
Micelles	POEG–VBC, creatine	Synergetic antitumor and antimetastatic activity Downregulation of antiapoptotic Bcl-2	-	miRNA prodrug and DOX	[143]
Nanocluster	Hyaluronic acid and BSA-coated gold nanocluster	Size-reducible DDS 95.3% suppression of in situ tumor growth and 88.4% inhibition of lung metastasis growth	-	PTX	[145]
Nanocluster	Hyaluronic acid-coated gold nanocluster	Size-reducible biomimetic nanoparticle Activation of CD4+, CD8+ T cells and NK cells and enhanced secretion of TNF-α and IL-12		PTX prodrug	[146]
Nano star	Chlorin e6 and PDA-coated gold nano star	Phototheranostic nanoprobe Production of singlet oxygen	-	-	[147]
Nanotube	Iron oxide NP-incorporated single-wall carbon nanotube	Magnetic (MRI)-driven theranostic nanoprobe	Anti-CD105	DOX	[148]
